# Fluidized Bed Membrane Reactor for the Direct Dehydrogenation of Propane: Proof of Concept

**DOI:** 10.3390/membranes12121211

**Published:** 2022-11-30

**Authors:** Camilla Brencio, Luca Di Felice, Fausto Gallucci

**Affiliations:** 1 Inorganic Membranes and Membrane Reactors, Sustainable Process Engineering, Department of Chemical Engineering and Chemistry, Eindhoven University of Technology, P.O. Box 513, 5600 MB Eindhoven, The Netherlands; 2 Eindhoven Institute for Renewable Energy Systems (EIRES), Eindhoven University of Technology, P.O. Box 513, 5600 MB Eindhoven, The Netherlands

**Keywords:** propane dehydrogenation, Pd membranes, hydrogen permeation, fluidized bed membrane reactors

## Abstract

In this work, the fluidized bed membrane reactor (FBMR) technology for the direct dehydrogenation of propane (PDH) was demonstrated at a laboratory scale. Double-skinned PdAg membranes were used to selectively remove H_2_ during dehydrogenation tests over PtSnK/Al_2_O_3_ catalyst under fluidization. The performance of the fluidized bed membrane reactor was experimentally investigated and compared with the conventional fluidized bed reactor (FBR) by varying the superficial gas velocity over the minimum fluidization velocity under fixed operating conditions (i.e., 500 °C, 2 bar and feed composition of 30vol% C_3_H_8_-70vol% N_2_). The results obtained in this work confirmed the potential for improving the PDH performance using the FBMR system. An increase in the initial propane conversion of c.a. 20% was observed, going from 19.5% in the FBR to almost 25% in the FBMR. The hydrogen recovery factor displayed a decrease from 70% to values below 50%, due to the membrane coking under alkene exposure. Despites this, the hydrogen extraction from the reaction environment shifted the thermodynamic equilibrium of the dehydrogenation reaction and achieved an average increase of 43% in propylene yields.

## 1. Introduction

With a processing capacity of 107.9 MMT in 2020 that is expected to reach 128 MMT by 2027, propylene is one of the most important raw materials in the petrochemical industry [1]. The growth in propylene production is primarily driven by the industry demand for polypropylene, which is used in a wide variety of applications, such as food packaging, electronics, and construction [2]. The increasing global demand for propylene over the past few years has given rise to the need for the development of more efficient conversion technologies and alternative feedstocks [3]. In this respect, Propane Dehydrogenation (PDH) is emerging as an attractive alternative to traditional processes [4]. In this process, propylene is directly produced from propane according to the following dehydrogenation reaction:

$$C_{3}H_{8}↔C_{3}H_{6}+H_{2},\;{\Delta H}_{R}^{298K}=124.3\;\mathrm{kJ}/\mathrm{mol}$$

Since the dehydrogenation reaction is reversible and endothermic, it is often carried out at high temperatures (550–650 °C) and atmospheric pressures using a platinum or chromium catalyst. Platinum offers a superior activation of the paraffinic C-H bond and low activity to C-C cleavage [4]. The activity towards dehydrogenation reactions originates from its metallic (non-oxidative) state. It has been proven that a single platinum site is able to break the C-H bond in propane [5]. The dehydrogenation reaction of light paraffins is insensitive to the structure of the Pt-particles: both the exposed crystallographic plane and the particle size do not influence the reaction. Only the number of active sites is relevant to the reaction, which means that small particles are preferred over larger particles. Platinum-based dehydrogenation catalysts are typically supported on alumina, due to its high thermal and mechanical stability, its high surface area, and its uniform pore size distribution. These two last features make is possible to maintain the platinum nanoparticles well dispersed on the support, which is crucial to attain stable catalyst performance [6]. A critical aspect of alumina supports is represented by their acidity, which promotes skeletal isomerization, cracking, and polymerization of olefinic materials, to enhance coke formation, which rapidly deactivates the catalyst. Therefore, alkaline metals, such as Li, Na and K, are added to poison the acidic sites of the support. Furthermore, olefins react faster on platinum than paraffins, due to the interaction activity between olefins and Pt being stronger than that of paraffins. The addition of platinum modifiers weakens the Pt–olefin interaction selectively, without affecting the Pt–paraffin interaction. Arsenic, tin, or germanium are among the metals reported as being platinum activity modifiers. They also improve catalyst stability against fouling by heavy carbonaceous materials [7]. Although Pt-based dehydrogenation catalysts are highly optimized, the process still suffers from side cracking reactions, which are favored by the high dehydrogenation temperatures and the consequent carbon formation, which rapidly deactivates the catalyst. These restrictions lead to great challenges to the dehydrogenation technologies [8]. The emerging technology of palladium-based membrane reactors shows a high degree of process intensification for the direct dehydrogenation of propane, and has demonstrated significant advantages over the conventional process [9,10,11,12]. The selective removal of H_2_ from the catalytic bed shifts the equilibrium beyond the thermodynamic limitations. In this way, it would be possible to achieve the same propane conversion as in the conventional reactor, but while working at lower operating temperatures, which results in a drastic reduction of the reactor heat duty, as well as in a reduction of the coke formation rate. Ricca et al. [13] carried out modeling and experimental work on a novel proposed process scheme for the direct dehydrogenation of propane, where Pd-based membranes used for the recovery of hydrogen were integrated in the fixed-bed reaction unit. Their results showed that when using membranes in the reaction unit, the propane conversion increased above its thermodynamic limitation, which also corresponded to the limit of a conventional reaction unit (i.e., without the membranes). Such a solution allows for a higher stability of the catalyst and, accordingly, a prolonged operation time that decreases the need for catalyst regeneration. Didenko et al. [14] experimentally investigated the dehydrogenation of propane in a combined packed-bed membrane reactor. In their work, they demonstrated at a laboratory scale the potential of the membrane reactor technology in increasing the feedstock conversion to propylene by a factor of 1.6–2.0 with respect to the conventional equilibrium value, under optimized operating conditions. Another interesting work that investigates the use of the membrane reactor technology for dehydrogenation processes is presented by He et al. [15]. In this work, the authors performed a thermodynamic analysis of a novel solar driven propane dehydrogenation system with a membrane reactor. The results showed that, compared to the performance of a conventional reactor (without membranes), an H_2_ permeate pressure of 10^−5^ bar increased the conversion rate of C_3_H_8_ from 4.1% to 99.12% and the selectivity of C_3_H_6_ from 93.1% to 99.1% at 400 °C. Thus, the membrane reactor has the potential to significantly increase the dehydrogenation reaction yield at lower temperatures by means of H_2_ separation.

Most of the scientific research on H_2_ selective membranes for dehydrogenation reactions has investigated the packed-bed membrane reactor (PBMR) configuration. This configuration offers, as main advantages, the simplicity in construction and the presence of consolidated and validated models for its design and scale-up. Moreover, packed-bed configurations avoid any damage of the membrane surface due to erosion from catalytic particles, since they are kept in a fixed position. However, the application of an integrated packed-bed membrane reactor for dehydrogenation processes is limited by the inevitable formation of carbon side products, which tend to adsorb on the surface of catalyst particles and membranes and subsequently dissociate, leading to their deactivation. Even though the coking of both the catalyst particles and the H_2_-selective membranes has been demonstrated to be reversible through regeneration with diluted oxygen, this requires different conditions: a hot air stream at 700 °C is needed to regenerate the catalyst, while Pd-based membranes can only be exposed to air up to 400 °C without compromising their surfaces. Compared to packed beds, the fluidized-bed membrane reactor (FBMR) allows for the movement of solid particles. When connected to an additional regeneration unit, in a Dual Circulating Fluidized Bed Membrane Reactor (DCFBMR) configuration, it becomes possible to have circulation of the solid particles from the reaction zone to the regeneration zone, so that the catalytic bed is always active and separately regenerates the catalyst and the membranes at the required operating conditions. Thus, the Dual Circulating Fluidized Bed Membrane Reactor offers a valuable solution for the catalyst regeneration, which is one of the main limitations of the PDH system. Prior to the evaluation of its industrial potential application for PDH processes, it is worth focusing on the fluidized bed membrane reactor configuration. The performance of the direct dehydrogenation of propane in a fluidized bed membrane reactor is experimentally investigated in this work. The aim is to demonstrate, at a laboratory scale, the higher efficiency of a membrane assisted fluidized bed reactor for the direct dehydrogenation of propane. Firstly, the fluidized bed membrane reactor technology is experimentally studied and compared with the conventional fluidized bed reactor (FBR) technology, with particular attention to the performance and stability of the membranes. Then, the experimental results are used to validate a one-dimensional phenomenological model for the FBMR. This allows us to extrapolate simulation results at a larger scale, which in turn can help in designing the DCFBMR configuration and get insights on its potential application at industrial scales.

## 2. Phenomenological Fluidized Bed (Membrane) Reactor Model Description

The model used in this work is based on the work of Iliuta et al. [16] that was later used by Medrano et al. [17]. The authors proposed a 1D continuum model describing the three-phase system of a bubbling bed model derived by Kunii and Levenspiel [18]. Both the gas and the solid phases are described in this model. The model for the gas flow consists of three phases: bubble, wake, and emulsion. The gas phase is fed into the reactor at a superficial gas velocity above the minimum fluidization velocity. The gas forms a bubble phase, with fraction f_b_ and with a characteristic bubble diameter d_b_ as functions of the axial position, which flows upwards with a velocity u_b_. The bubble carries with its movement a fraction of solid in the so-called wake phase. The volumetric fraction of wake α is estimated to be 15% of the bubble volume. The remaining gas moves upwards in the emulsion phase at the emulsion velocity u_e_. Mass transfer occurs between the gas in the emulsion phase and the gas in the bubble phase, with a mass transfer coefficient K_be_. On the other hand, the solids present a net downward flow in the emulsion phase with an emulsion velocity u_se_, and they are exchanged with solids in the wake-bubble phase with a mass transfer coefficient K_we,s_. A summary of the hydrodynamics and mass transfer correlations obtained from the literature and used in this work is reported in Appendix A, Table A1.

For the gas phase, it is possible to write mass balances both in the bubble and in the emulsion phase, as reported in Equations (1) and (2).

(1) $$\frac{d}{\mathrm{dz}}\left[u_{b}f_{b}\left(1+f_{w}e_{\mathrm{mf}}\right)C_{i,\mathrm{bw}}\right]=\pm R_{i,\mathrm{bw}}f_{\mathrm{bw}}\left(1-e_{\mathrm{mf}}\right)+K_{i,\mathrm{be}}\left(f_{b}+f_{w}e_{\mathrm{mf}}\right)\left(C_{i,\mathrm{ce}}-C_{i,w}\right)$$

(2) $$\frac{d}{\mathrm{dz}}\left[u_{g,e}\left(f_{\mathrm{ce}}e_{\mathrm{mf}}\right)C_{i,\mathrm{ce}}\right]=\pm R_{i,\mathrm{ce}}f_{\mathrm{ce}}\left(1-e_{\mathrm{mf}}\right)-K_{i,\mathrm{be}}\left(f_{b}+f_{w}e_{\mathrm{mf}}\right)\left(C_{i,\mathrm{ce}}-C_{i,w}\right)$$

The balances are each solved per different instants of time at each axial position of the bed. Similarly, for the solid phase, the model solves the mass balances for every axial position in the wake and in the emulsion phases, as presented in Equations (3) and (4).

(3) $$\frac{d}{\mathrm{dz}}\left[u_{b}f_{w}\left(1-e_{\mathrm{mf}}\right)C_{i,w}\right]=\pm R_{i,w}f_{w}\left(1-e_{\mathrm{mf}}\right)+K_{i,\mathrm{we}}f_{w}\left(1-e_{\mathrm{mf}}\right)\left(C_{i,\mathrm{ce}}-C_{i,w}\right)$$

(4) $$\frac{d}{\mathrm{dz}}\left[u_{\mathrm{se}}f_{\mathrm{ce}}\left(1-e_{\mathrm{mf}}\right)C_{i,\mathrm{ce}}\right]=\pm R_{i,\mathrm{ce}}f_{\mathrm{ce}}\left(1-e_{\mathrm{mf}}\right)-K_{i,\mathrm{we}}f_{w}\left(1-e_{\mathrm{mf}}\right)\left(C_{i,\mathrm{ce}}-C_{i,w}\right)$$

Overall, two solid phases are modeled in this work: the fresh and the deactivated catalyst, on top of which coke is deposited. Commercial PtSnK/Al_2_O_3_ particles, which have been extensively investigated in the literature for the dehydrogenation of propane and for deriving gas–solid reaction rate expressions to model coke formation [19,20,21,22], are considered as catalytic material in the model. The direct dehydrogenation of propane is described by the following reaction scheme:
$C_{3}H_{8}↔C_{3}H_{6}+H_{2}$  
${\Delta H}_{R}^{298K}=124.3\;\mathrm{kJ}/\mathrm{mol}$
(R1)$C_{3}H_{8}\;↔{\mathrm{CH}}_{4}+C_{2}H_{4}$  
${\Delta H}_{R}^{298K}=98.9\;\mathrm{kJ}/\mathrm{mol}$
(R2)$C_{2}H_{4}+H_{2}\;↔C_{2}H_{6}$  
${\Delta H}_{R}^{298K}=-136.6\;\mathrm{kJ}/\mathrm{mol}$
(R3)$C_{3}H_{6}\;↔3{\mathrm{CH}}_{0.5}+2.25H_{2}$  
${\Delta H}_{R}^{298K}=119.5\;\mathrm{kJ}/\mathrm{mol}$
(R4)

The heterogenous catalyzed gas-phase reactions R1–R3 and gas–solid reaction R4 are assumed to follow the kinetics of Lobera et al. [7]. The corresponding reaction rate expressions are reported in Equations (5)–(8).

(5) $$-r_{1}=\alpha \frac{k_{1}\left(P_{C3H8}-\left(\frac{P_{C3H6}P_{H2}}{K_{\mathrm{eq}}}\right)\right)}{1+\left(P_{C3H8}K_{C3H6}\right)}$$

(6) $$-r_{2}=k_{2}P_{C3H8}$$

(7) $$-r_{3}=k_{3}P_{C2H4}P_{H2}$$

(8) $$\frac{\mathrm{dC}}{\mathrm{dt}}k_{1C}{\left(C_{\max}-C_{m}\right)}^{2}+k_{2c}$$

To account for the catalyst deactivation due to carbon formation (R4), the parameter α is multiplied by the propane dehydrogenation rate expression r_1_. This parameter correlates the activity with the content of coke on the catalyst, and it is expresses as:

(9) $$\alpha =\left(1-\gamma_{1}C_{m}\right)+\gamma_{2}C_{m}\exp\left(-\gamma_{3}\left(\frac{C_{M}}{C_{m}}\right)\right)$$

Where C_m_, C_M_, and C_max_ are the coke concentration in the monolayer, in the multilayer, and the maximum coke concentration in the monolayer, respectively. The expression of all the kinetic parameters present in the equations listed above are reported in Appendix A.1. The PDH reactor model developed in this work can be simulated as a conventional fluidized bed reactor (PDH-FBR), and as a fluidized bed membrane reactor (PDH-FBMR), in which a dead-end Pd-based membrane is integrated to selectively remove hydrogen from the reactor. The hydrogen flux permeating through the Pd-based membrane is described by the following equation (Equation (10)):

(10) $$J_{H_{2}}=\alpha \left(t\right)\frac{1}{\delta }{\mathrm{Pe}}_{0}\exp\left(-\frac{E_{a}}{\mathrm{RT}}\right)\left(p_{H_{2}\;\mathrm{ret}}^{n}-p_{H_{2}\mathrm{perm}}^{n}\right)$$

where Pe_0_ is the pre-exponential of the membrane permeability, E_a_ is the activation energy, δ is the membrane thickness, p_H2 ret_ and p_H2 perm_ are the hydrogen partial pressure at the retentate and permeate side, respectively, and n is the pressure exponent. The hydrogen flux expression is multiplied by the dimensionless transient deactivation term α(t), which correlates the activity of the Pd-based membranes to selectively separate hydrogen with the carbon content formed on the membrane surface that is responsible for its deactivation over time. In this way, it is possible to model the hydrogen flux decay over time, which is representative of the membrane deactivation trend. The membrane activity coefficient α(t) has been derived by the same authors in a previous work [23], and its expression, together with the values of the kinetic parameters, are reported in Appendix A.1. The main permeation parameters used in this modeling work are reported in Table 1, and result from the fitting of the pure hydrogen permeation experiments, as already described by the same authors in a previous work [24].

The hydrogen flux permeating through the membrane is split in emulsion and bubble phases that are proportional to their fractions in the bed. To account for the removal of hydrogen from the reaction ambient, the flow rate of hydrogen permeated through the membrane, calculated from the definition of the hydrogen flux (Equation (10)), is subtracted from the differential mass balances of hydrogen compounds along the reactor length for both the bubble and the emulsion phases.

The resulting system of partial differential equations has been discretized using a simple first order upwind finite difference scheme for the convection terms, which is solved using the Newton–Raphson’s iterative method.

## 3. Materials and Methods

### 3.1. Fluidized Bed (Membrane) Reactor Setup

**Figure 1:**
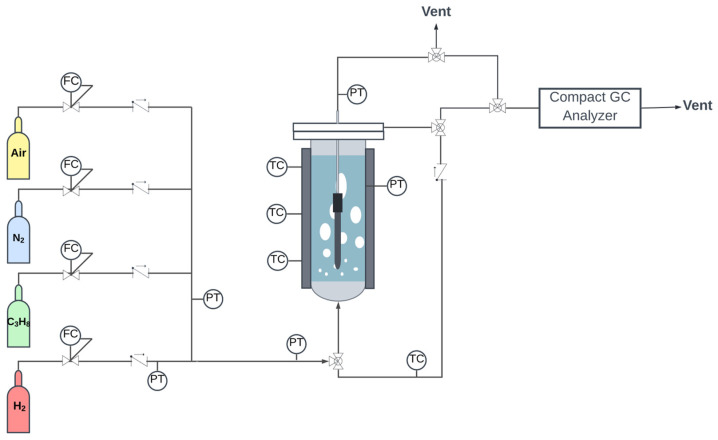
Schematic representation of the experimental setup for fluidization tests. FC indicates mass flow controllers, TC represents thermocouples, and PT indicates pressure transducers.

A schematic representation of the setup used for both the fluidized bed reactor and the fluidized bed membrane reactor experiments is shown in Figure 1.

The setup consists of a stainless-steel reactor with an internal diameter of 0.043 m and a bed height of 0.44 m, and with a porous plate distributor made of Hastelloy X (40 μm pore size). The reactor is placed in an electrically heated oven to maintain isothermal conditions. The temperature was measured by three thermocouples placed inside the reactor bed, one at the bottom, one in the middle, and the last one at the top. The flow rate of the process gases was regulated by Bronkhorst digital mass flow controllers. A back-pressure controller was installed to regulate the operating pressure in the reaction side. The reactor system can be operated both as a conventional fluidized bed reactor and as a fluidized bed membrane reactor by opening/closing the permeate line. The permeate side of the membrane was either operated at atmospheric pressure or at a vacuum pressure. An automated soap bubble flow meter from Horibastec, with a measuring range of 0–0.2 LSTP/min, was used to measure the volumetric flow rate at the permeate side. The composition of both the retentate and the permeate streams was analyzed with a compact gas chromatograph GC (Global Analyzer Solution TM, G.A.S., Breda, The Netherlands) equipped with a TCD detector and three packed columns (HayeSep Q 60–80 mesh and 5A molecular sieve) for the analysis of permanent gases (i.e., H_2_, CO_2_, CO and N_2_) and an FID detector with capillary columns (Rtx-1, MTX-1 and MTX-QBond) was used for the analysis of the hydrocarbons.

### 3.2. H_2_-Selective Membrane

A novel type of Pd-alloyed membrane, known as a double-skinned membrane, was used in this work. This membrane was prepared following the procedure described by Arratibel at el. [25]. A thin Pd and Ag selective layer was deposited by the electroless plating technique onto a Al_2_O_3_ porous tubular substrate, with an external/internal diameter of 14/7 mm and an external pore size of 100 nm provided by Rauschert Kloster Veilsdorf. After the Pd–Ag layer deposition, the sample was turned into a double-skinned membrane by placing an additional thin (<1 μm) mesoporous ceramic (50wt% YSZ- 50wt% γ-Al_2_O_3_) layer on top of the Pd–Ag layer through a vacuum-assisted dip coating technique at room temperature. The morphology and the chemical composition of the double-skinned membrane were determined by Scanning Electron Microscope (SEM) and Energy Dispersive X-Ray (EDX) analysis, using a FEI Quanta 250 FEG equipment.

**Figure 2:**
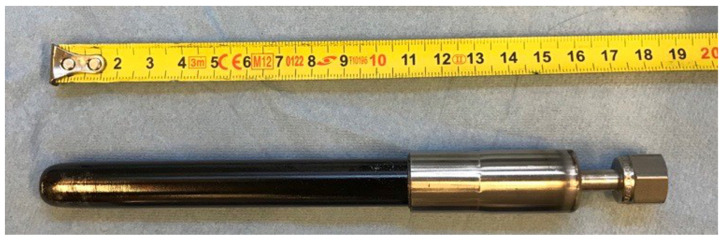
Double-skinned membrane with a fingerlike configuration and the other end sealed.

The additional ceramic layer has already been demonstrated to have a protective effect against mechanical erosion of the selective Pd–Ag layer by particle attritions in fluidized beds by Arratibel et al. [26], and it was proven for the direct dehydrogenation of propane in this work. The membrane has one end closed (i.e., fingerlike configuration), while the other end was sealed with graphite ferrules and a metallic connector using a hydraulic crimping machine (FINN-POWER), as shown in Figure 2.

The membrane, with a total active length of 11 cm, was integrated from the top flange of the reactor with a stainless-steel tube, leaving a distance of 5 cm between the bottom plate distributor and the closed end of the membrane. Prior to the catalytic tests, membrane stability tests were performed to assess the absence of any chemical interaction between the catalyst particles and the membrane selective layer. The membrane was exposed for almost 7 h under a gas mixture of 60vol% H_2_ and 40vol% N_2_, in a continuous bubbling fluidization regime, working at 500 °C with 1 bar of pressure difference across the membrane.

During the reaction tests, the membrane performance was monitored by measuring the hydrogen recovery factor, defined as:

(11) $$H_{2}\;\mathrm{Recovery}\;\mathrm{Factor}=\frac{F_{H_{2},\mathrm{permeate}}}{F_{H_{2},\mathrm{retentate}}+F_{H_{2},\mathrm{permeate}}}$$

### 3.3. Catalyst

The catalyst used in this work was prepared following the procedure reported in the literature [27]. A sequential wet impregnation procedure was used to synthetize the Pt-Sn-K catalyst, which was impregnated onto a 160–250 μm γ-Al_2_O_3_ supplied by Sasol. The resulting nominal composition of the catalyst was 0.05, 0.14, and 0.10wt% of Pt, Sn, and K, respectively (Table 2). The specific surface area (S.A.) and pore volume (P.V.) were determined via the BET and BJH elaboration of the N_2_ adsorption–desorption isotherms at −196 °C, obtained using a Micromeritics ASAP 2020 gas adsorption device. Before the measurement, the sample was degassed at 250 °C for 2 h. The morphology and the chemical composition of the fresh catalyst were analyzed by Scanning Electron Microscope (SEM) and Energy Dispersive X-Ray (EDX) analysis, using a FEI Quanta 250 FEG equipment. The catalyst reducibility was studied via temperature programmed reduction (TPR) analysis performed using a Micromeritics AutoChem 2920 equipment with a TCD detector. The analysis was carried out in the range of 100–700 °C with a heating rate of 10 °C·min^−1^, while feeding 50 mL·min^−1^ of a 10% H_2_/Ar mixture. Prior to the TPR analysis, the sample was outgassed under inert conditions for the N_2_ physisorption. X-ray diffraction (XRD) analysis in the 2θ range of 10–120° was performed on the reduced catalyst with a MiniFlex600 machine (Rigaku) operating with a Ni β-filtered Cu-Kα radiant at 40 kV and 30 mA and a scan step of 0.05°/min. The results of these characterization techniques are reported in Appendix A.2.

To cover the full active membrane surface under gas–solid suspension at minimum fluidization velocity, the catalyst was diluted with inter alumina in a mixture of 195 g of particles (10wt%/90wt%). Blank tests were conducted on both the empty stainless steel reactor and on the reactor filled solely with γ-Al_2_O_3_ particles to confirm the absence of any activity towards the dehydrogenation of propane of both the reactor walls and the filler particles, as reported in Appendix A.3.

Prior to the catalytic fluidization tests, the catalyst was exposed to 5 consecutive cycles of reduction-reaction-oxidation to make the catalyst stable and aged [7]. The catalyst pre-treatment was repeated after each reactive test as a reference to assess the stability and the reusability of the catalyst. The experimental conditions and results of those tests are reported in Appendix A.3. The minimum fluidization velocity was determined experimentally for the filler particles at different temperatures and atmospheric pressures using the standard pressure drop method [28]. According to this method, the volumetric flow rate at the inlet of the reactor is varied in a range of 0–100 mL/min and the pressure difference along the reactor bed is measured by a pressure drop transducer that reaches up to 50 mbar. The minimum fluidization velocity is found when the pressure difference in the reactor is constant. The results reported in Table 3 were used to select the feed flow rate for the reactive experiments necessary to keep the catalyst bed in the bubbling fluidization regime.

## 4. Results and Discussion

### 4.1. H_2_-Selective Membrane Characterization

**Figure 3:**
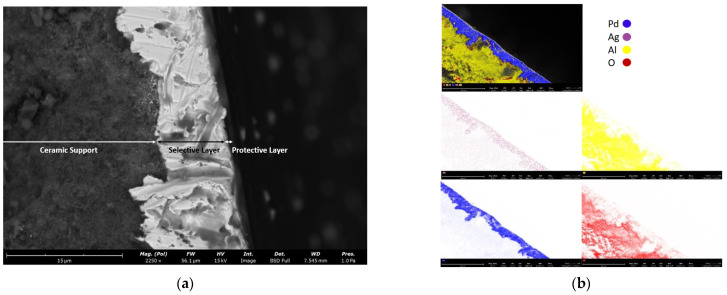
SEM (**a**) and EDX (**b**) image of the cross-section of the double-skinned Pd–Ag membrane used in this work.

The different layers of the double-skinned Pd–Ag membrane and their chemical compositions were determined by Scanning Electron Microscope (SEM) and Energy Dispersive X-Ray (EDX) analysis. Figure 3 shows the cross-section image of the membrane. Looking at Figure 3a from the left to the right, it is possible to distinguish the ceramic support (left), the dense selective layer (middle) and the thin protective ceramic layer (right).

**Figure 4:**
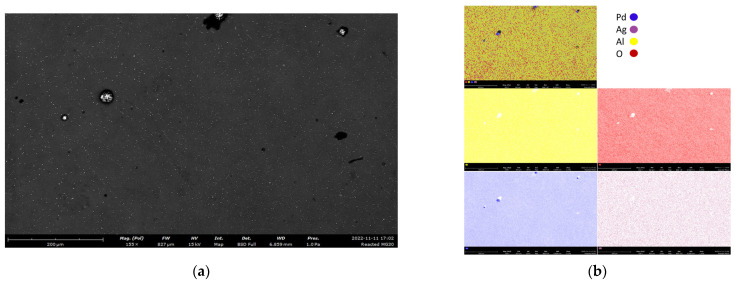
SEM (**a**) and EDX (**b**) image of the top layer of the double-skinned Pd–Ag membrane used in this work.

The darker areas in Figure 3a mainly consist of oxygen and aluminum (Figure 3b), which represent the protective ceramic layer. The lighter areas show the presence of palladium and silver (Figure 3b), which was detected with a high atomic ratio of 24:1. This is an indication of the hydrogen selective Pd–Ag layer. The SEM-EDX of the top protective layer is reported in Figure 4. In this case, oxygen and aluminum were retrieved over the whole surface with high atomic concentrations of 65.6% and 25.6%, respectively, which indicated the presence of γ-Al_2_O_3_ in the top ceramic protective layer, while palladium and silver of the underlying selective layer were detected in lower amounts (7.6% and 1.2%, respectively).

### 4.2. Preliminary Simulation Study to Identify Optimum Operative Conditions

Prior to the experimental activities, a simulation study was carried out using the phenomenological reactor model described in Section 2. The aim of this study was to identify the optimum operating conditions to be used at a laboratory scale for propane dehydrogenation reaction tests. The criteria were calculated to maximize propane conversion and thus to be able to appreciate any difference between the FBR and the FBMR configurations under this regime. The simulations were performed by modeling the reaction unit with same geometry and catalyst bed composition used for the experimental activities, as described in Section 3. The effects of temperature, feed composition, and pressure on both propane conversion and carbon concentration, expressed in kg_coke_/kg_cat_, were investigated, and the total feed flow rate was set to have a superficial gas velocity over minimum fluidization velocity ratio equal to 3.75. It is worth mentioning that the simulations were performed for the conventional fluidized bed reactor configuration, while the comparison between the FBR and the FBMR were investigated experimentally with the chosen operating conditions.

**Figure 5:**
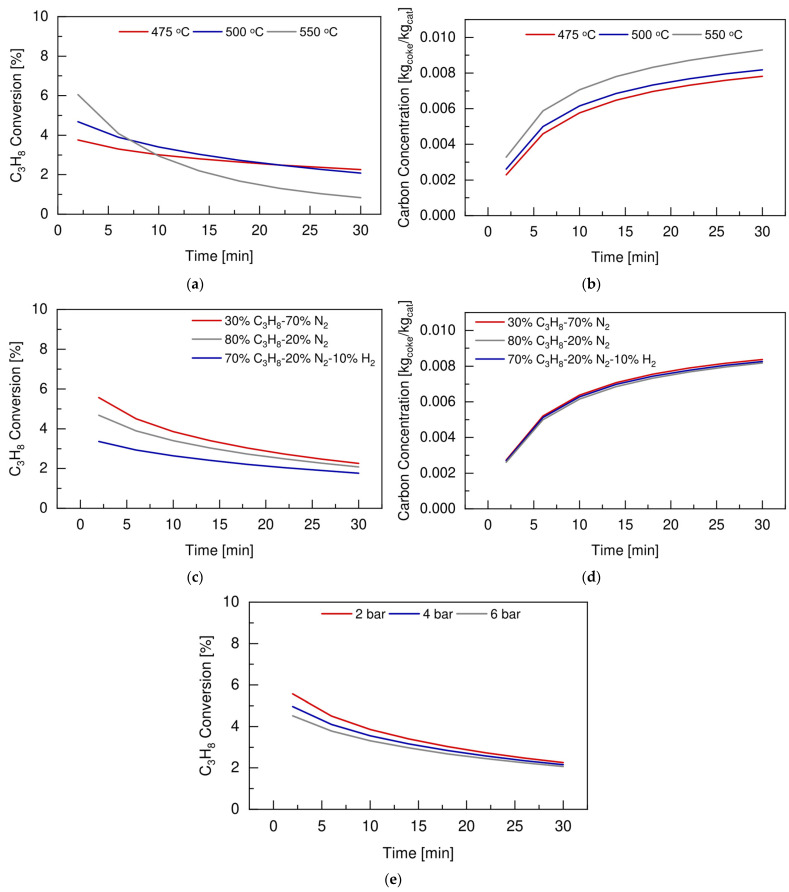
Effect of reaction temperature on (**a**) propane conversion and (**b**) carbon concentration, at 2 bar, u/u_mf_ = 3.75 and feed composition of 80vol%C_3_H_8_ and 20vol%N_2_. Effect of feed composition on (**c**) propane conversion and (**d**) carbon concentration, at 500 °C, 2 bar, u/u_mf_ = 3.75. Effect of operating pressure (**e**) on propane conversion, at 500 °C, u/u_mf_ = 3.75 and feed composition of 30vol%C_3_H_8_ and 70vol%N_2_.

Figure 5a,b shows the effects of temperature on propane conversion and carbon concentration, respectively, at 2 bar and using a feed composition of 30vol% C_3_H_8_ and 70vol% N_2_.

These preliminary results show that the higher the operating temperature, the higher the initial propane conversion, due to the endothermic nature of the system. However, the evolution of the conversion with time displays a faster decrease at higher temperature. Propane conversion drops of c.a. 40% occurred at 475 °C, while it experienced a reduction up to 86.2% at 550 °C after 30 min. This can be explained by the more severe catalyst deactivation, since the higher the temperature increased, the higher the carbon concentration was (Figure 5b).

The effects of the feed composition were investigated at 500 °C and 2 bar, and the results in terms of propane conversion and coke deposition are reported in Figure 5c,d, respectively.

These results show that, with only 30vol% of propane in the feed, it was possible to increase the initial propane conversion by almost 10% when compared to the case in which the feed contained 80 vol% of reagent. This would not affect the catalyst deactivation much, as shown by the similar carbon concentration formed as a function of the vol% of propane in the feed. On the other hand, when hydrogen is also present in the feed composition (i.e., blue lines), it acted as a diluent that lowered the final coke concentration by 2.28% when compared to the case of 30vol% of propane in the feed. At the same time, the presence of hydrogen in the feeding mixture reduced the overall propane conversion due to the nature of the main dehydrogenation reaction (R1) being limited by the thermodynamic equilibrium. Finally, the effects of the operating pressure were investigated at 500 °C under a feed composition of 30vol% C_3_H_8_ and 70vol% N_2_. As shown in Figure 5e, the higher the operating pressure, the lower the propane conversion was, due to the thermodynamic nature of the main dehydrogenation reaction. Based on the above reported considerations, a temperature of 500 °C, a pressure of 2 bar, and a feed composition of 30vol% C_3_H_8_ and 70vol% N_2_ were identified as the optimum operating conditions to perform propane dehydrogenation reaction tests under fluidization at a laboratory scale.

### 4.3. Conventional Fluidized Bed Reactor

**Figure 6:**
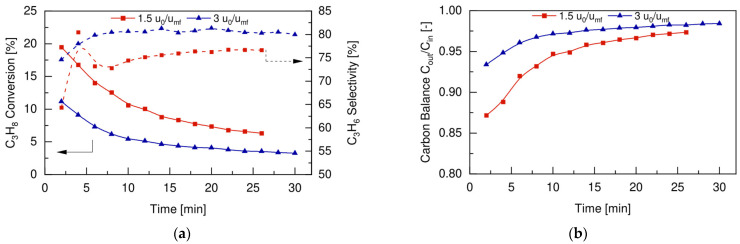
Fluidized bed reactor (**a**) performance and (**b**) carbon balance for different u_0_/u_mf_ ratios, at 500 °C, 1 bar and with a feed composition of 30vol% C_3_H_8_-70vol% N_2_.

Figure 6a shows the experimental results in terms of propane conversion and propylene selectivity as a function of time obtained for the catalytic fluidized bed reactor (without membrane) for the direct dehydrogenation of propane at two different superficial gas velocities over minimum fluidization velocity ratios (u_0_/u_mf_), at 500 °C and 2 bar.

The higher is the ratio of u_0_/u_mf_, the faster the inlet gas was and the lower the total residence time in the reactor was. As expected, propane conversion increased for lower u_0_/u_mf_, since the gas had longer time to be in contact with catalyst particles and, as a consequence, to be converted. However, this led to higher carbon formation due to the faster catalyst deactivation. This can be observed in Figure 6b, which reports the carbon balance over time, expressed according to the following equation (Equation (12)):

(12) $$C\;\mathrm{balance}=\frac{C_{\mathrm{out}}}{C_{\mathrm{in}}}=\frac{\sum_{i}n_{i}F_{i,\mathrm{out}}}{\sum_{i}n_{i}F_{i,\mathrm{in}}}$$

where i indicates all the components with carbon atoms, n_i_ is the number of carbon atoms, and F_i_ is the molar flow rate.

Moreover, a lower selectivity towards propylene (Figure 6a) was obtained for the lower u_0_/u_mf_ ratio, since the cracking reactions prevailed over the main dehydrogenation reaction for longer residence times. Therefore, for a fixed reaction temperature, an increase in the inlet flow rate will result in a reduced C_3_H_8_ conversion rate but at the same time will allow to reach higher propylene selectivity by reducing the total amount of carbon formed during the dehydrogenation process.

### 4.4. Fluidized Bed Membrane Reactor

#### 4.4.1. Membrane Stability Tests

**Figure 7:**
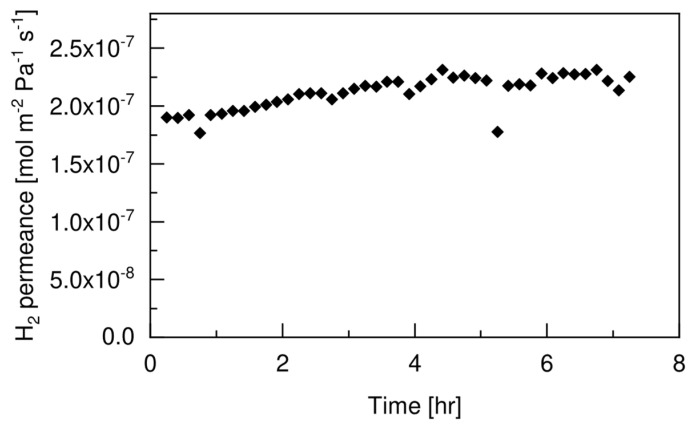
Membrane performance over time under fluidization conditions at 500 °C, ΔP = 2 bar, u_0_/u_mf_ = 5 and feeding a mixture of 60 vol% H_2_ and 40 vol% N_2_.

The presence of the protective layer on top of the selective Pd-based layer of the double-skinned membrane used in this work has already been demonstrated to improve the mechanical stability under long-term (~600 h) fluidization conditions [26]. With this test, we aimed at verifying the absence of any chemical interaction between the selective Pd-based layer of the membrane and the Pt-based catalyst particles. For this purpose, a short term permeation test of hydrogen was sufficient, since a chemical interaction between the palladium and the catalyst used in the test would lead to a sharp drop in the performance of the membrane after several minutes, as already observed by Fernandez et al. [29] and Okazaki et al. [30]. Figure 7 shows the stability performance of the double-skinned membrane, in terms of hydrogen permeance, under fluidization conditions for almost 6 h.

The H_2_ permeance of the membrane showed an initial increasing trend (first hour of test), due to the activation of the Pd-selective layer under exposure to hydrogen. Afterward, the membrane reached a plateau with a value of hydrogen permeance of 2.1 mol m^−2^ Pa^−1^ s^−1^. The membrane did not suffer from any decay in the hydrogen permeance with time. This confirmed the absence of any chemical interaction between the Pt-based catalyst and the membrane surface.

#### 4.4.2. Reaction Tests

**Figure 8:**
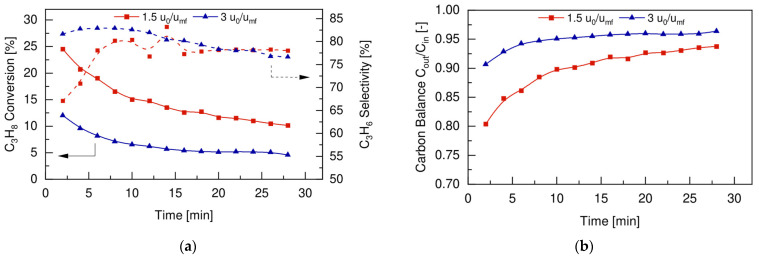
Fluidized bed membrane reactor (**a**) performance and (**b**) carbon balance for different u_0_/u_mf_ ratios, at 500 °C, 2 bar and with a feed composition of 30vol% C_3_H_8_-70vol% N_2_.

Once the membrane stability under fluidization conditions was verified, the fluidized bed membrane reactor concept for the PDH was experimentally proven. The reaction tests were conducted under similar operating conditions as those used for the conventional fluidized bed PDH reactions to maintain consistency and comparability between the results. The effects of the superficial gas velocity over minimum fluidization velocity ratios (u_0_/u_mf_) on the FBMR performance were investigated at 500 °C, with an inlet feed composition of 30vol% C_3_H_8_ and 70vol% N_2_, working at 2 bar in the retentate side and using a vacuum in the permeate side. The reactor performance in terms of propane conversion, propylene selectivity, and carbon balance are reported in Figure 8a,b, respectively.

As already observed in the conventional FBR (see Section 4.2), propane conversion increased with the lower u_0_/u_mf_ ratio, since the reactive mixture had longer contact time to convert with catalyst particles. At the same time, the activity initially decreased faster at lower u_0_/u_mf_ ratios, again due to a faster coke formation, as shown in Figure 8b. The main advantage of using the FBMR configuration is represented by the different trend obtained for the propylene selectivity, compared to the one shown in Figure 8a for the FBR. When hydrogen was extracted from the catalytic bed, the equilibrium was shifted to the product side, and thus the propylene selectivity increased at the beginning and then stabilized to constant values of c.a. 78% over time. Even though initially the propylene selectivity was lower at the lower u_0_/u_mf_ ratio, it increased faster with respect to the case of the higher u_0_/u_mf_ ratio. This is because hydrogen extraction makes the dehydrogenation reaction predominant over cracking side reactions. Thus, the use of a FBMR configuration for PDH allowed for work at a lower inlet gas flow rate, which reached a higher C_3_H_8_ conversion rate and higher propylene selectivity, and overcame the trade-off between conversion and selectivity that is typical of the FBR configuration (see Figure 6a).

### 4.5. FBR vs. FBMR and Model Validation

**Figure 9:**
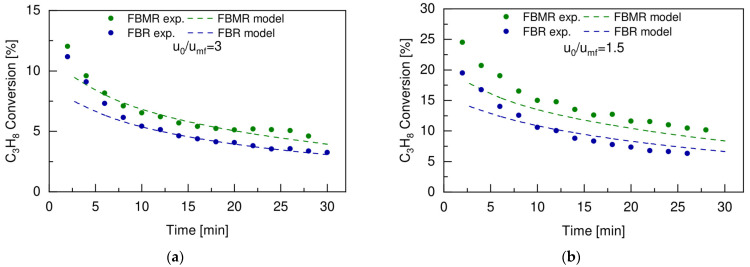
Propane conversion versus time obtained experimentally (dots) and with model simulation (dashed lines) for the FBR and the FBMR, working at 500 °C, 2 bar, with a feed composition of 30vol% C_3_H_8_-70vol% N_2_, with (**a**) u_0_/u_mf_ = 3 and (**b**) u_0_/u_mf_ = 1.5.

In this section, the performance of the conventional fluidized bed reactor is compared with the ones of the fluidized bed membrane reactor obtained under the same operating conditions. In particular, the conversion of propane (expressed in mol%) obtained at 500 °C and 2 bar, with a feed composition of 30vol% C_3_H_8_ and 70vol% N_2_ for two different u_0_/u_mf_ ratios, is compared for the FBR and the FBMR configurations. The experimental results were compared with modeling results to validate the 1D phenomenological model at different operating conditions. Figure 9a,b shows the results obtained from both experiments and model simulations with u_0_/u_mf_ =3 and u_0_/u_mf_ =1.5, respectively.

In general, the model described the experimental results quite well for the two different u_0_/u_mf_ considered, and for both the FBR and the FBMR configurations. Deviations between ±10–20% were observed for the first experimental points within the first 5 min of the test. This could be related to the fact that the catalyst particles at the beginning showed a higher reactivity under the experimental conditions used in this test compared to the one predicted by the model. The average deviations in the model predictions compared with the experimental data were below 10%. Therefore, it can be concluded that the phenomenological model has been validated and that it can adequately predict the performance of the PDH system in a fluidized bed membrane reactor. However, this model was developed under the assumption of neglecting radial dispersions. Since lateral concentration profiles can affect the overall reactor performance, additional work is needed to account for those additional phenomena and make the model more robust. This will allow it to be used for the design and scale-up of the fluidized bed membrane reactor for PDH, as well as for the fundamental understanding of all the phenomena that are involved. This will help to identify the main limitations of the system and to consequently investigate the DCFBMR configuration as a reaction intensification strategy.

**Figure 10:**
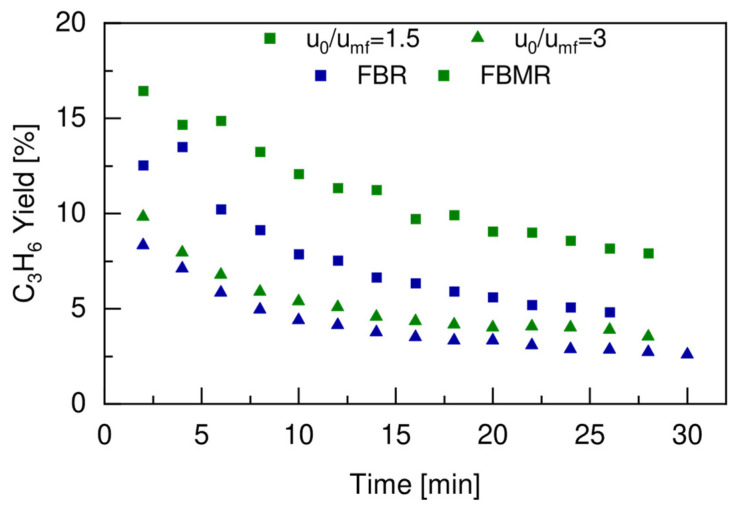
Propylene yield in the FBR (blue symbols) and FBMR (green symbols) working at 500 °C, 2 bar, with a feed composition of 30vol% C_3_H_8_-70vol% N_2_, for u_0_/u_mf_ = 1.5 (■) and u_0_/u_mf_ = 3 (▲).

As already observed in the previous section, the higher C_3_H_8_ conversion was obtained when working at the lower u_0_/u_mf_ for both the FBR and the FBMR. For this reason, the main advantage of utilizing hydrogen selective membrane (FBMR) to circumvent the thermodynamic equilibrium limitation was obtained at the lower u_0_/u_mf_ ratio, as is clearly shown in Figure 9b. This would result, at the same time, in a higher total flow rate of hydrogen produced during the dehydrogenation reaction, and therefore it would have a bigger impact on the shift in the thermodynamic equilibrium when extracted through the H_2_ selective membrane. The initial propane conversion could be increased by almost 21%, going from 19.5% in the FBM to 24.5% in the FBMR, when working with a u_0_/u_mf_ ratio of 1.5, resulting in an average increase of almost 32% for the entire reaction time compared to the average increment of 19% that was obtained working with a u_0_/u_mf_ ratio equal to 3. The positive effect of removing hydrogen to shift the equilibrium toward propylene was confirmed by the trends of the propylene yield over time, which are reported in Figure 10 for both the FBR and the FBMR at the two different u_0_/u_mf_ ratios tested in this work. The propylene yield was always higher in the FBMR than in the FBR configuration, and this was even more evident for a lower u_0_/u_mf_ ratio, which reached an average increase of 43% at a ratio of 1.5 u_0_/u_mf_ compared to the one obtained at a ratio of 3 u_0_/u_mf_, which was equal to 24%.

The performance of PDH under fluidization obtained in this work are compared with the ones reported in the literature. Most of the scientific works have been conducted in packed-bed reactor (PBR) configurations with the integration of membranes, but in only a few of them was the fluidized bed membrane reactor technology investigated. The propane conversion, operating conditions, catalyst, and reactor configuration used are summarized in Table 4.

**Figure 11:**
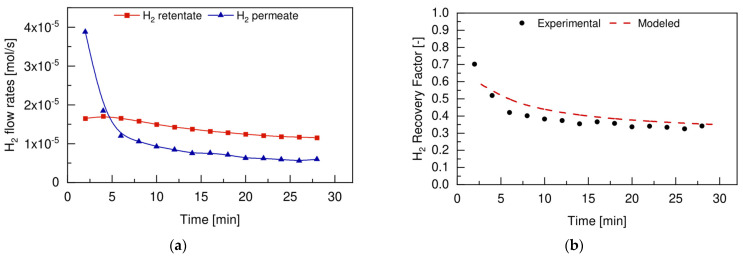
(**a**) Hydrogen flow rates measured in the retentate and permeate side of the FBMR and (**b**) the corresponding hydrogen recovery factor, measured at 500 °C, 2 bar, u_0_/u_mf_ of 1.5, and with a feed composition of 30vol% C_3_H_8_-70vol% N_2_.

During the test performed with u_0_/u_mf_ equal to 1.5, the performance of the membrane was monitored by measuring the total flow rate permeated and by analyzing its composition. The total flow rate of hydrogen in the permeate side was compared with the total flow rate of hydrogen produced in the retentate side during the dehydrogenation reaction, as shown in Figure 11a. The corresponding hydrogen recovery factor is reported in Figure 11b.

**Figure 12:**
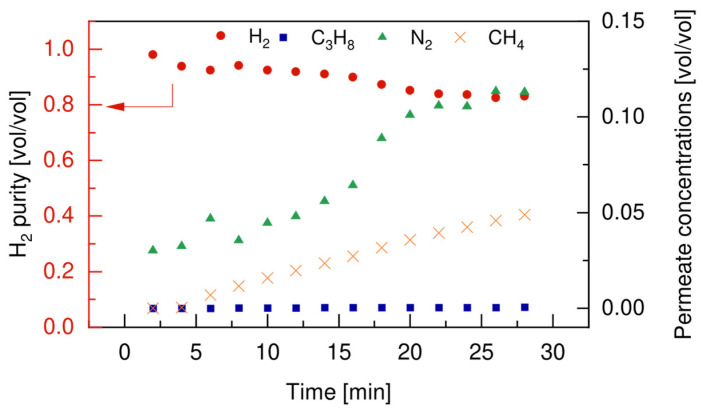
Permeate composition measured at 500 °C, 2 bar, u_0_/u_mf_ of 1.5, and with a feed composition of 30vol% C_3_H_8_-70vol% N_2_.

The total amount of hydrogen produced during the dehydrogenation reaction (red line in Figure 11a) had a decreasing trend over time, which could be attributed to the continuous extraction of hydrogen through the membrane, but also to the reduced activity of the catalyst. On the other hand, a typical transient deactivation trend of the hydrogen permeated through the membrane, due to the deactivation of the Pd-based membrane when exposed to alkenes, as already reported by other authors. This made the hydrogen recovery factor go from an initial value of 70.2% to a value of 40.1% in the first 5 min of the test, with a resulting average H_2_ recovery factor of 40%. Even though the hydrogen recovery factor followed a decreasing trend and stabilized at values around 40%, the main advantage of using the FBMR technology can still be attributed to the positive effect of hydrogen removal from the reaction ambient. This led to a higher conversion of propane towards propylene (see Figure 10), which reduced the extent of side cracking products formation, such as ethane, ethylene, and methane, for which the selectivity in the FBMR is reduced. These observations were also confirmed by the analysis of the permeated outlet stream compositions, which reported in Figure 12.

The GC did not detect any C_3_H_8_ in the permeate side of the system, which was attributed to the shift in the thermodynamic equilibrium of the main dehydrogenation reaction to the extraction of hydrogen. For this component, the vol% detected in the permeate side was almost constant in the first 15 min of test, while it started to decrease in the remaining part of the test. This can be attributed to the membrane coking, as confirmed by the continuous growing vol% of methane in the permeate side. The presence of small traces of other components, i.e., N_2_ and CH_4_, in the permeate site could be attributed to defects on the membrane sealing, which increased under operation.

## 5. Conclusions

In this work, we demonstrated at a laboratory scale the fluidized bed membrane reactor technology for the direct dehydrogenation of propane. We analyzed the performance of the FBMR configuration, and we compared it with the conventional FBR for different u_0_/u_mf_ ratios. According to the results obtained, propane conversion was increased when working at lower u_0_/u_mf_ ratios in both the FBR and FBMR, which gave higher contact time to the catalyst particles to interact with the reactive mixture. This resulted in a reduced propylene selectivity in the FBR, since the cracking reactions became predominant over the main dehydrogenation reaction for longer residence times. On the contrary, the hydrogen extraction in the FBMR made the dehydrogenation reaction predominant over cracking side reactions for longer residence times. This led to an increase of almost 21% in propane conversion when working at the lower u_0_/u_mf_ ratio, which went from 19.5% in the FBM to 24.5% in the FBMR. These experimental results confirmed the main advantage of utilizing the hydrogen selective membrane (FBMR) to circumvent the thermodynamic equilibrium limitation, despite the limited performance of the Pd-based membrane used. The FBMR system for PDH can be further improved by mitigating the coking behavior of Pd-based membranes, for which an average hydrogen recovery factor below 50% was experimentally measured during the tests. This would require the development of improved Pd-based membrane configurations. Finally, the experimental results validated the 1D phenomenological model developed in this work, which observed an overall discrepancy lower than 10% for the propane conversion. From here, we can conclude that the model can be used to predict the behavior of the FBMR at different operating conditions and scales, and most importantly it can be used to further investigate the Dual Circulating Fluidized Bed Reactor configuration. This in its turn can help in optimizing and designing scaled-up experimental versions of this reactor concept.

## Figures and Tables

**Table 1:** Permeability parameters for the Pd–Ag membrane used in this work.

Parameter	Units	Value
Pe_0_	mol/(m^2^ s Pa)	5.73 × 10^−10^
E_a_	kJ/mol	12.53
δ	μm	1.75
n	-	0.75

**Table 2:** Catalyst and filler particles physical properties.

Material	D_p_ [μm]	Avg. Skeletal Density [g/cm^3^]	Apparent Density [g/cm^3^]	W [g]
Al_2_O_3_	150–250	3.300	1.691	175
0.05 PtSnK/Al_2_O_3_	200–250	3.317	1.204	20

**Table 3:** Experimental values of minimum fluidization velocity as function of temperature.

Parameter	Value		
Temperature [°C]	20	400	500
u_mf_ [m/s]	0.036	0.009	0.008

**Table 4:** Experimental values of minimum fluidization velocity as function of temperature.

Reactor Configuration	Catalyst	T [°C] and p [bar]	Feed Composition [vol%]	WHSV [h^−1^] or u_0_/u_mf_	Conversion [%]	Ref.
PBR	Pt-Sn/γAl_2_O_3_	580, 1	80% C_3_H_8_-20% H_2_	5 h^−1^	25%	[22]
PBMR	Pt-Sn based	500, 6	80% C_3_H_8_-20% H_2_O	8 h^−1^	<8%	[13]
FBR	Pt-Sn-K/γAl_2_O_3_	500, 1	50% C_3_H_8_-50% Ar	4	<10%	[31]
FBR	Pt-Sn/Al-SAPO-34	590, 1	80% C_3_H_8_-20% H_2_	9 h^−1^	30%	[32]
FBR	CrO_x_-based	550, 1	100 C_3_H_8_	2.49 h^−1^	25%	[33]
FBMR	Pt-Sn/MgAl_2_O_4_	525, 1	50% C_3_H_8_-50% Ar	2.5	15%	[34]
FBR	Pt-Sn-K/γAl_2_O_3_	500, 2	30% C_3_H_8_-70% N_2_	1.5	19%	This work
FBMR	Pt-Sn-K/γAl_2_O_3_	500, 2	30% C_3_H_8_-70% N_2_	1.5	24.5%	This work

**Figure A1:**
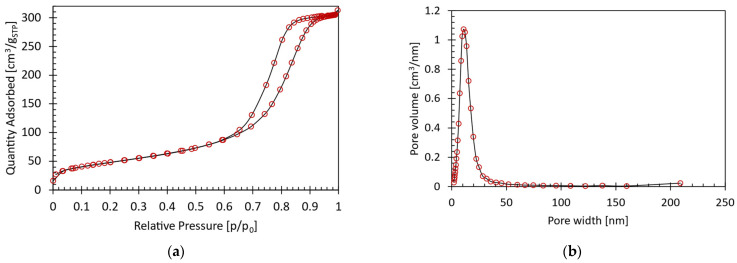
Volume of N_2_ adsorbed/desorbed as a function of the relative pressure (P/P_0_) (**a**) and pore volume as a function of the pore width (**b**), as derived from the BJH elaboration of the desorption branch.

**Figure A2:**
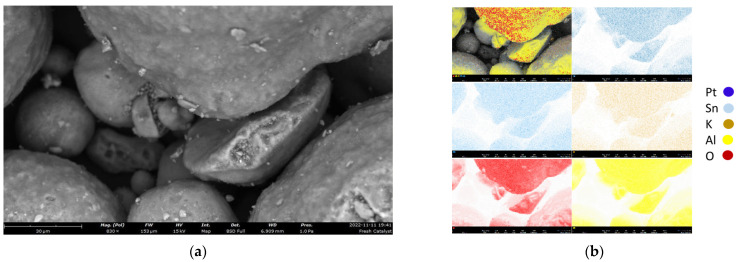
SEM (**a**) and EDX (**b**) images of the fresh PtSnK/Al_2_O_3_ catalyst used in this work.

**Figure A3:**
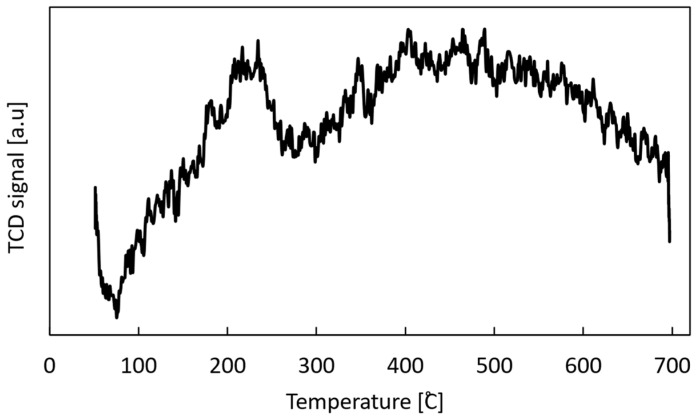
TPR signal obtained via TPR analysis of the fresh Pt-Sn-K/Al_2_O_3_ catalyst used in this work.

**Figure A4:**
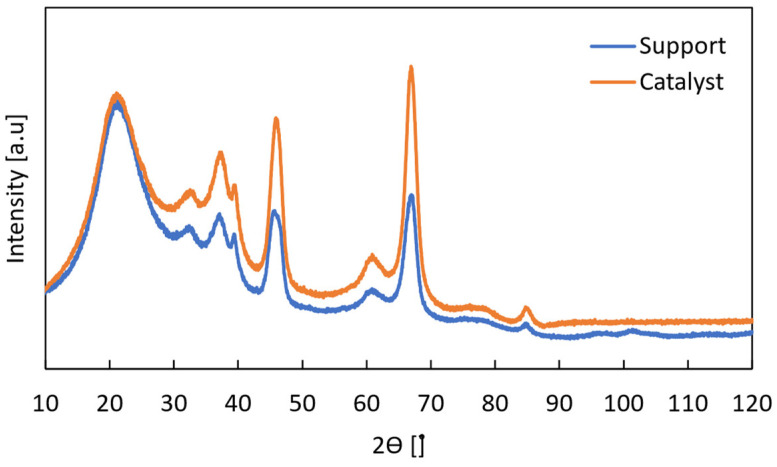
XRD spectra of the fresh catalyst (orange line) and the support (blue line) samples.

**Figure A5:**
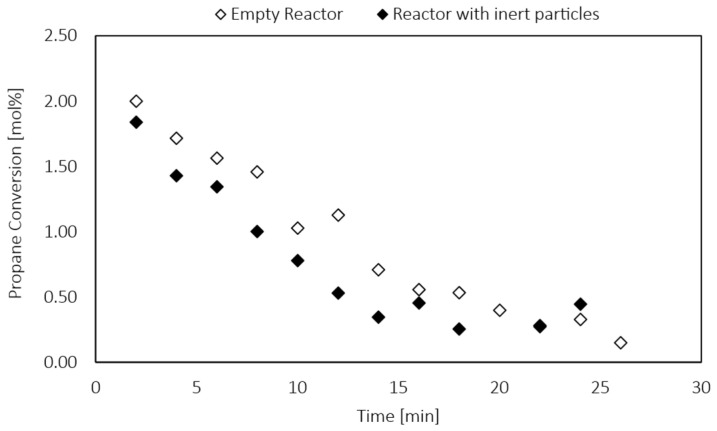
Blank test results for the empty reactor (empty dots) and the reactor filled with inert particles (full dots) at 500 °C, atmospheric pressure, and feeding a mixture of 30vol% C_3_H_8_-70vol% N_2_.

**Figure A6:**
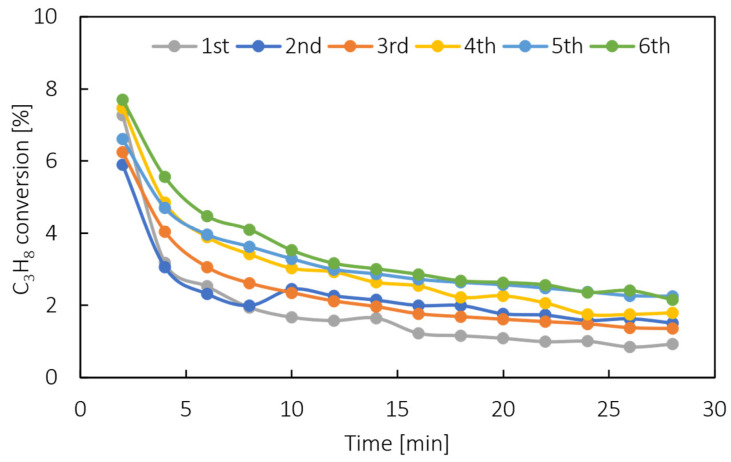
Propane conversion versus time for each reactive cycle performed for the catalyst stabilization (first 6 cycles).

**Figure A7:**
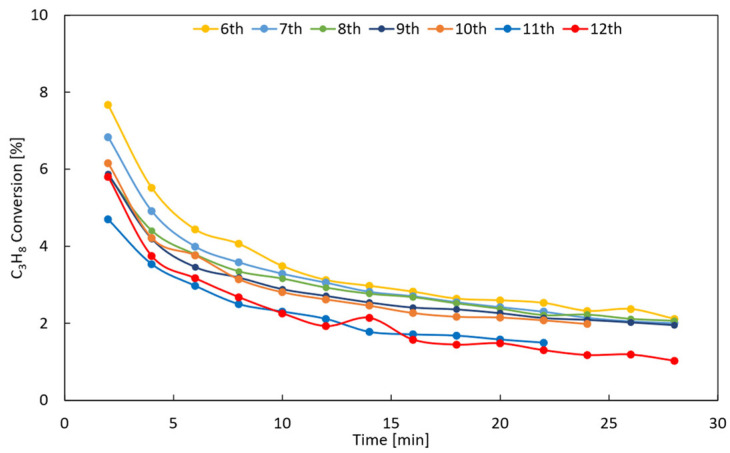
Propane conversion versus time for each reactive cycle performed for the catalyst stabilization (for larger amount of cycles).

## Data Availability

Data will be made available upon request.

## Appendix A

The table below reports the closure equations used for the modeling of the fluidized bed reactor.

**Table A1:** Empirical correlations used for hydrodynamics and mass transfer.

Parameter	Equation	Ref.
Archimedes number	$Ar=\frac{d_{p}^{3}\rho_{g}\left(\rho_{p}-\rho_{g}\right)g}{\mu_{g}^{2}}$	[35]
Minimum fluidization velocity	$u_{mf}=\frac{\mu_{g}}{\rho_{g}d_{p}}\sqrt{{\left(27.2\right)}^{2}+0.0408Ar-27.2}$	
Bed void fraction at minimum fluidization	$e_{mf}=0.586Ar^{-0.029}{\left(\frac{\rho_{p}}{\rho_{g}}\right)}^{0.021}$	[36]
Bubble dimensions	$d_{b,0}=0.376{\left(u_{0}-u_{mf}\right)}^{2}$ $d_{b,max}=0.65\left(\frac{\pi }{4}D_{T}^{2}{\left(u_{0}-u_{mf}\right)}^{0.4}\right)$ $d_{b}=d_{b,max}-\left(d_{b,max}-d_{b,0}\right)e^{-\frac{0.3h}{D_{T}}}$	[18,37]
Bubble fraction	$f_{b}\approx \frac{u_{0}-u_{mf}}{u_{b}}$	[18]
Wake fraction	$f_{w}=\alpha \;f_{b}\;$	[38]
Emulsion fraction	$f_{ce}=1-f_{b}-f_{w}$	
Bubble velocity	$u_{b}=u_{0}-u_{mf}+0.711{\left(g-d_{b}\right)}^{0.5}$	[18]
Gas emulsion velocity	$u_{e}=\frac{u_{0}-\left(f_{b}+f_{w}e_{mf}\right)u_{b}}{f_{ce}e_{mf}}$	
Solid Emulsion velocity	$u_{s,e}=\frac{f_{w}\left(1-e_{mf}\right)u_{b}+\frac{feed}{A}}{f_{ce}\left(1-e_{mf}\right)}$	
Mass transfer coefficient gas phase	$K_{i,bc}=4.5\frac{u_{mf}}{d_{b}}+5.85\frac{D_{g}^{0.5}g^{0.25}}{d_{b}^{1.25}}$ $K_{i,ce}=6.77{\left(\frac{D_{g}\epsilon_{mf}u_{b}}{d_{b}^{3}}\right)}^{0.5}$ $K_{i,be}=\frac{1}{K_{bc}}+\frac{1}{K_{ce}}$	
Mass transfer coefficient solid phase	$K_{i,we}=\frac{0.0075\left(u_{0}-u_{mf}\right)}{u_{mf}d_{b}}\;\;if\frac{u_{0}}{u_{mf}}\leq 3$ $K_{i,we}=\frac{0.15}{d_{b}}\;if\frac{u_{0}}{u_{mf}}>3\;$	

### Appendix A.1. Reaction Rate Laws and Kinetic Parameters

The kinetic model used for the simulation of the PDH reactor was based on the reaction pathways proposed in the work of Lobera et al. [7], for which the kinetic parameters are reported in Table A2.

**Table A2:** List of kinetic parameters for the Pt-Sn-K/Al_2_O_3_ catalyst [7].

Kinetic Parameter Expressions	Parameter	Value	Units
$k_{1}=k_{01}exp\left(\frac{-E_{a1}}{R}\left(\frac{1}{T}-\frac{1}{T_{m}}\right)\right)$ $K_{c3H6}=K_{0}exp\left(\frac{-\Delta H}{R}\left(\frac{1}{T}-\frac{1}{T_{m}}\right)\right)$	$k_{01}$ $E_{a1}$ $K_{0}$ ΔH $T_{m}$	0.5242 34.57 3.46 −85.817 500	${\mathrm{mmol}\;g}^{-1}{\;\min}^{-1}{\mathrm{bar}}^{-1}$ ${\mathrm{kJmol}}^{-1}$ - ${\mathrm{kJmol}}^{-1}$ °C
$k_{2}=k_{02}exp\left(\frac{-E_{a2}}{R}\left(\frac{1}{T}-\frac{1}{T_{m}}\right)\right)$	$k_{02}$ $E_{a2}$	0.00465 137.31	${\mathrm{mmol}\;g}^{-1}{\;\min}^{-1}{\mathrm{bar}}^{-1}$ ${\mathrm{kJmol}}^{-1}$
$k_{3}=k_{03}exp\left(\frac{-E_{a3}}{R}\left(\frac{1}{T}-\frac{1}{T_{m}}\right)\right)$	$k_{03}$ $E_{a3}$	0.000236 154.54	${\mathrm{mmol}\;g}^{-1}{\;\min}^{-1}{\mathrm{bar}}^{-1}$ ${\mathrm{kJmol}}^{-1}$
$C_{m}=C_{MAX}^{2}\left[\frac{k_{1c}t}{1+C_{MAX}k_{1c}t}\right]$ $C_{M}=k_{2c}t$ $k_{1c}=k_{01c}exp\left(\frac{-E_{a1c}}{R}\left(\frac{1}{T}-\frac{1}{T_{m}}\right)\right)$ $k_{2c}=k_{02c}exp\left(\frac{-E_{a2c}}{R}\left(\frac{1}{T}-\frac{1}{T_{m}}\right)\right)$	$k_{01c}$ $E_{a1c}$ $k_{01c}$ $E_{a1c}$ $C_{MAX}$	234 38.43 1.45 × 10^−6^ 125.51 1.04 × 10^−3^	${\mathrm{mg}}_{\mathrm{cat}}/{\mathrm{mg}}_{\mathrm{coke}}\min$ ${\mathrm{kJmol}}^{-1}$ ${\mathrm{mg}}_{\mathrm{coke}}/{\mathrm{mg}}_{\mathrm{cat}}\min$ ${\mathrm{kJmol}}^{-1}$ ${\mathrm{mg}}_{\mathrm{coke}}/{\mathrm{mg}}_{\mathrm{cat}}$

In order to account for the catalyst deactivation, the parameter α is multiplied by the propane dehydrogenation rate expression r_1. This parameter correlates the activity with the content of coke on the catalyst; according to the model proposed by Lobera et al., a simple mechanistic model called the monolayer-multilayer coke growth model (MMCGM) is used to describe coke formation. Both the monolayer and the multilayer of coke formed are responsible for catalyst deactivation, and this is proven by the experimental activity time results, which show that activity decreases even after the monolayer has been fully formed.

As a result, the catalyst activity is expresses as:

(A1) $$a=\left(1-\gamma_{1}C_{m}\right)+\gamma_{2}C_{m}\exp\left(-\gamma_{3}\left(\frac{C_{M}}{C_{m}}\right)\right)$$

(A2) $$\gamma_{1}=\gamma_{01}\exp\left(-\frac{E_{a\gamma 1}}{R}\left(\frac{1}{T}-\frac{1}{T_{m}}\right)\right)$$

Where *C_m_*, *C_M_*, and *C_max_* are the coke concentration in the monolayer, in the multilayer and the maximum coke concentration in the monolayer, respectively. Table A3 reports the values of the kinetic parameters used in Equations (A1) and (A2).

**Table A3:** List of parameters for the catalyst deactivation [7].

Kinetic Parameter	Value	Units
$\gamma_{01}$	948.92	$g_{\mathrm{cat}}/g_{\mathrm{coke}}$
$E_{a\gamma 1}$	9.61	${\mathrm{kJmol}}^{-1}$
$\gamma_{2}$	399	$g_{\mathrm{cat}}/g_{\mathrm{coke}}$
$\gamma_{3}$	40.07	−

Similarly, the membrane activity is expressed as a function of the amount of carbon formed in the monolayer and in the multilayer, as follows:

(A3) $$\alpha \left(t\right)={\left(1-\alpha_{1}C_{m}-\alpha_{2}C_{M}\right)}^{m}$$

(A4) $$C_{m}=C_{max}-{\left(\left(1-h\right)k_{1c}t+C_{max}^{1-h}\right)}^{\frac{1}{1-h}}\;\;$$

(A5) $$C_{M}=k_{2c}t$$

(A6) $$k_{ic}=k_{ic,0}\exp\left(-\frac{E_{ia}}{R}\left(\frac{1}{T}-\frac{1}{T_{0}}\right)\right)\;\;$$

Table A4 reports the values of the kinetic parameters used in Equations (A3)–(A6).

**Table A4:** List of parameters for the Pd-based membrane deactivation [23].

Kinetic Parameter	Value	Units
$\;\alpha_{1}$	9.989	${\mathrm{cm}}_{\mathrm{membrane}}^{2}/{\mathrm{mg}}_{\mathrm{coke}}\;$
$\;\alpha_{2}$	2.221 × 10^−14^	${\mathrm{cm}}_{\mathrm{membrane}}^{2}/{\mathrm{mg}}_{\mathrm{coke}}\;$
m	1.557	−
$\;C_{max}$	0.096	${\mathrm{mg}}_{\mathrm{coke}}/{\mathrm{cm}}_{\mathrm{membrane}}^{2}$
h	5.1	−
$\;k_{1c,0}\;$	1.175e^3^	${\mathrm{cm}}_{\mathrm{membrane}}^{2}/{\left({\mathrm{mg}}_{\mathrm{coke}}\min\right)}^{1-h}\;$
$\;k_{2c,0}$	1.848 × 10^−9^	${\mathrm{mg}}_{\mathrm{coke}}/{\mathrm{cm}}_{\mathrm{membrane}}^{2}\min$
$\;E_{a1}$	1.822 × 10^5^	J/mol
$\;E_{a2}$	1.007 × 10^6^	J/mol
$\;T_{0}$	405	°C

### Appendix A.2. Physicochemical Properties of the PtSnK-Al_2_O_3_ Catalyst

Figure A1 reports the results of the N_2_ physisorption analysis in terms of the volume of N_2_ adsorbed vs. relative pressure (a), and pore volume as a function of the pore width (b), as derived from the BJH elaboration of the desorption branch.

A cumulative BET surface area of 263.2 m^2^/g was measured, with a total pore volume of 0.53 cm^3^/g and an average pore diameter of 10.7 nm.

Figure A2 reports the SEM-EDX images of the fresh catalyst.

From the SEM analysis of Figure A2a, it was not possible to distinguish the active phase and detect its morphology. Figure A2b confirms that most of the sample was made by Al_2_O_3_, which represents the support. Al and O were the elements with the highest atomic concentration, equal to 60.9% and 38.3%, respectively. The active phase of the catalyst was detected with lower atomic concertation, due to the low amount (<0.3wt%) present in the catalyst formulation. Overall, the EDX analysis reported an atomic concentration of Pt, K, and Sn equal to 0.02, 0.45, and 0.23%, respectively.

The catalyst reducibility was studied via temperature programmed reduction (TPR) analysis performed using a Micromeritics AutoChem 2920 equipment with a TCD detector. The analysis was carried out in the range 100–700 °C with a heating rate of 10 °C·min^−1^ feeding 50 mL·min^−1^ of a 10% H_2_/Ar mixture. Prior to the TPR analysis, the sample was outgassed under inert conditions for the N_2_ physisorption.

From the TPR signal reported in Figure A3, it was not possible to retrieve a clear H_2_ consumption profile as a function of temperature. The profile obtained during the analysis did not exhibit clear reduction peaks, due to the low amount of active metal Pt present on it (i.e., 0.05wt%).

The crystalline structure of both the Pt-Sn-K/γ-Al_2_O_3_ and the γ-Al_2_O_3_ support was analyzed via XRD spectra, which are depicted in Figure A4.

Both samples showed peaks at 2θ = 66.96°, 45.84°, 39.48°, 37.36°, and 19.2°, which are assignable to the (4 4 0), (4 0 0), (2 2 2), (3 1 1), and (1 1 1) planes of cubic γ-Al2O3, respectively, as reported in the literature [39]. No additional peaks were detected that could be attributed to the catalyst sample. Due to the very little amount of metal (<0.3wt%) present in the catalyst formulation, it was not possible to distinguish the crystalline structure of the catalyst in the diffractogram.

### Appendix A.3. Blank Tests

To assess if the walls of the reactor and/or the inert particles used to dilute the catalyst catalyzed the main dehydrogenation reaction, two blank tests were conducted at 500 °C at atmospheric pressure and under a feeding mixture of 30vol% C3H8 and 70vol% N2, which was representative of the reactive operating conditions used for PDH tests. The test was first conducted on the empty reactor and then on the reactor filled with inert particles, and the results are reported in Figure A5.

A very small amount of propane was converted, with an average conversion of 0.911% for the empty reactor, and 0.792% for the reactor with inert particles. Most importantly, no differences were observed from the two cases analyzed, which indicated that the small amount of propane conversion catalyzed by the wall of the reactor or by the inert packing could be neglected.

### Appendix A.4. Catalyst Stability Tests

The catalyst stabilization consisted of three cyclical steps, where the catalyst was first reduced with diluted H_2_ during 2 h, then PDH was carried out in the presence of diluted C_3_H_8_ (30 min), and finally, coke deposited over the catalyst surface was removed with a stream of diluted oxygen. The operating conditions of each step are summarized in Table A5.

**Table A5:** Experimental conditions during the stabilization cycles.

Operating Conditions					
Cycle	Temperature [°C]	Pressure [bar]	Feed Composition	Duration [h]	u_0_/u_mf_ [-]
Reduction	550	1	H_2_:N_2_ = 1:2	2	5
Reaction	500	1	C_3_H_8_:N_2_ = 2.33	0.5	
Oxidation	500	1	Air:N_2_ = 1:10	1	

This cyclical process was repeated until two consecutive results were identical, and this situation occurred after five consecutive cycles, as shown in Figure A6.

The 6th cycle was taken as a reference to evaluate the stability and, most importantly, the reusability of the catalyst, before starting a new reactive test.

As shown in Figure A7, it was possible to keep the catalyst stable over five reactive tests, each lasting 30 min. However, after the 5th reactive test, the results obtained during the pre-treatment cycle revealed that both the initial conversion and, most importantly, the overall trend over time decreased when compared with the previous cycles. This indicated that the catalyst was losing its activity. Thus, the Pt-Sn-K/Al_2_O_3_ catalyst can be used with stable properties for five consecutive reduction-reaction-oxidation steps, at the conditions reported in Table A5.

## References

[1] Global Industry Analysts Global Propylene Industry. https://www.reportlinker.com/p05799443/Global-Propylene-Glycol-Industry.html?utm_source=GNW.

[2] Saerens S., Sabbe M.K., Galvita V.V., Redekop E.A., Marin G.B. (2017). The Positive Role of Hydrogen on the Dehydrogenation of Propane on Pt (111). ACS Catal..

[3] Nawaz Z. (2015). Light Alkane Dehydrogenation to Light Olefin Technologies: A Comprehensive Review. Rev. Chem. Eng..

[4] Sattler J.J.H.B., Ruiz-Martinez J., Santillan-Jimenez E., Weckhuysen B.M. (2014). Catalytic Dehydrogenation of Light Alkanes on Metals and Metal Oxides. Chem. Rev..

[5] Martino M., Meloni E., Festa G., Palma V. (2021). Propylene Synthesis: Recent Advances in the Use of Pt-Based Catalysts for Propane Dehydrogenation Reaction. Catalysts.

[6] Vora B.V. (2012). Development of Dehydrogenation Catalysts and Processes. Top. Catal..

[7] Lobera M.P., Téllez C., Herguido J., Menéndez M. (2008). Transient Kinetic Modelling of Propane Dehydrogenation over a Pt-Sn-K/Al_2_O_3_ Catalyst. Appl. Catal. A Gen..

[8] Chen S., Chang X., Sun G., Zhang T., Xu Y., Wang Y., Pei C., Gong J. (2021). Propane Dehydrogenation: Catalyst Development, New Chemistry, and Emerging Technologies. Chem. Soc. Rev..

[9] Shelepova E.V., Vedyagin A.A. (2020). Intensification of the Dehydrogenation Process of Different Hydrocarbons in a Catalytic Membrane Reactor. Chem. Eng. Process. Process Intensif..

[10] Shelepova E.V., Vedyagin A.A., Mishakov I.V., Noskov A.S. (2015). Simulation of Hydrogen and Propylene Coproduction in Catalytic Membrane Reactor. Int. J. Hydrog. Energy.

[11] Wang H., Wang B., Qi X., Wang J., Yang R., Li D., Hu X. (2021). Innovative Non–Oxidative Methane Dehydroaromatization via Solar Membrane Reactor. Energy.

[12] Shelepova E.V., Vedyagin A.A., Mishakov I.V., Noskov A.S. (2011). Mathematical Modeling of the Propane Dehydrogenation Process in the Catalytic Membrane Reactor. Chem. Eng. J..

[13] Ricca A., Montella F., Iaquaniello G., Palo E., Salladini A., Palma V. (2019). Membrane Assisted Propane Dehydrogenation: Experimental Investigation and Mathematical Modelling of Catalytic Reactions. Catal. Today.

[14] Didenko L.P., Savchenko V.I., Sementsova L.A., Chizhov P.E., Bykov L.A. (2013). Dehydrogenation of Propane in a Combined Membrane Reactor with Hydrogen-Permeable Palladium Module. Pet. Chem..

[15] He R., Wang Y., Wang H., Lundin S.T.B., Wang B., Kong H., Lu X., Wang J., Li W. (2021). A Mid/Low-Temperature Solar-Driven Integrated Membrane Reactor for the Dehydrogenation of Propane—A Thermodynamic Assessment. Appl. Therm. Eng..

[16] Iliuta I., Tahoces R.S., Patience G. (2010). Chemical-Looping Combustion Process: Kinetics and Mathematical Modeling. AIChE J..

[17] Medrano J.A., Potdar I., Melendez J., Spallina V., Pacheco-Tanaka D.A., van Sint Annaland M., Gallucci F. (2018). The Membrane-Assisted Chemical Looping Reforming Concept for Efficient H2 Production with Inherent CO2 Capture: Experimental Demonstration and Model Validation. Appl. Energy.

[18] Levenspiel O., Kunii D. (1991). Fluidization Engineering.

[19] Li Q., Sui Z., Zhou X., Chen D. (2011). Kinetics of Propane Dehydrogenation over Pt-Sn/Al_2_O_3_ Catalyst. Appl. Catal. A Gen..

[20] Zangeneh F.T., Taeb A., Gholivand K., Sahebdelfar S. (2013). Kinetic Study of Propane Dehydrogenation and Catalyst Deactivation over Pt-Sn/Al_2_O_3_ Catalyst. J. Energy Chem..

[21] Sahebdelfar S., Ravanchi M.T., Tahriri Zangeneh F., Mehrazma S., Rajabi S. (2012). Kinetic Study of Propane Dehydrogenation and Side Reactions over Pt-Sn/Al_2_O_3_ Catalyst. Chem. Eng. Res. Des..

[22] Farjoo A., Khorasheh F., Niknaddaf S., Soltani M. (2011). Kinetic Modeling of Side Reactions in Propane Dehydrogenation over Pt-Sn/γ-Al_2_O_3_ Catalyst. Sci. Iran..

[23] Brencio C., Gough R., de Leeuw den Bouter A., Arratibel A., Di Felice L., Gallucci F. (2023). Kinetic Model for Pd-Based Membranes Coking/Deactivation in Propane Dehydrogenation Processes. Chem. Eng. J..

[24] Brencio C., Fontein F.W.A., Medrano J.A., Felice L., Di Arratibel A., Gallucci F. (2021). Pd-Based Membranes Performance under Hydrocarbon Exposure for Propane Dehydrogenation Processes: Experimental and Modeling. Int. J. Hydrog. Energy.

[25] Arratibel A., Pacheco A., Laso I., Sint M. (2018). Van Development of Pd-Based Double-Skinned Membranes for Hydrogen Production in Fl Uidized Bed Membrane Reactors. J. Memb. Sci..

[26] Arratibel A., Medrano J.A., Melendez J., Pacheco Tanaka D.A., van Sint Annaland M., Gallucci F. (2018). Attrition-Resistant Membranes for Fluidized-Bed Membrane Reactors: Double-Skin Membranes. J. Memb. Sci..

[27] Bariås O.A., Holmen A., Blekkan E.A. (1996). Propane Dehydrogenation over Supported Pt and Pt–Sn Catalysts: Catalyst Preparation, Characterization, and Activity Measurements. J. Catal..

[28] Helmi A., Fernandez E., Melendez J., Tanaka D.A.P., Gallucci F., Van Sint Annaland M. (2016). Fluidized Bed Membrane Reactors for Ultra Pure H2 Production—A Step Forward towards Commercialization. Molecules.

[29] Fernandez E., Helmi A., Coenen K., Melendez J., Viviente J.L., Pacheco Tanaka D.A., Van Sint Annaland M., Gallucci F. (2015). Development of Thin Pd-Ag Supported Membranes for Fluidized Bed Membrane Reactors Including WGS Related Gases. Int. J. Hydrog. Energy.

[30] Okazaki J., Ikeda T., Pacheco Tanaka D.A., Llosa Tanco M.A., Wakui Y., Sato K., Mizukami F., Suzuki T.M. (2009). Importance of the Support Material in Thin Palladium Composite Membranes for Steady Hydrogen Permeation at Elevated Temperatures. Phys. Chem. Chem. Phys..

[31] Lobera M.P., Téllez C., Herguido J., Menéndez M. (2008). Propane Dehydrogenation over Pt-Sn-K/γ-Al_2_O_3_ Catalyst in a Two-Zone Fluidized Bed Reactor. Ind. Eng. Chem. Res..

[32] Nawaz Z., Chu Y., Yang W., Tang X., Wang Y., Wei F. (2010). Study of Propane Dehydrogenation to Propylene in an Integrated Fluidized Bed Reactor Using Pt-Sn/Al-SAPO-34 Novel Catalyst. Ind. Eng. Chem. Res..

[33] Song C., Wang J., Wang S., Wen J. (2022). Experimental and Theoretical Study of the Impact of Operating Conditions on Catalytic Propane Dehydrogenation in a Fluidized Bed Reactor. Ind. Eng. Chem. Res..

[34] Medrano J.A., Julián I., Herguido J., Menéndez M. (2013). Pd-Ag Membrane Coupled to a Two-Zone Fluidized Bed Reactor (TZFBR) for Propane Dehydrogenation on a Pt-Sn/MgAl2O4 Catalyst. Membranes.

[35] Lippens B.C., Mulder J. (1993). Prediction of the Minimum Fluidization Velocity. Powder Technol..

[36] Broadhurst T.E., Becker H.A. (1975). Onset of Fluidization and Slugging in Beds of Uniform Particles. AIChE J..

[37] Mori S., Wen C.Y. (1975). Estimation of Bubble Diameter in Gaseous Fluidized Beds. AIChE J..

[38] Medrano J.A., Tasdemir M., Gallucci F., van Sint Annaland M. (2017). On the Internal Solids Circulation Rates in Freely-Bubbling Gas-Solid Fluidized Beds. Chem. Eng. Sci..

[39] He S., Sun C., Bai Z., Dai X., Wang B. (2009). Dehydrogenation of Long Chain Paraffins over Supported Pt-Sn-K/Al_2_O_3_ Catalysts: A Study of the Alumina Support Effect. Appl. Catal. A Gen..

